# Wogonoside alleviates metabolic dysfunction-associated steatohepatitis by modulating AMPK and xCT/GPX4 pathways to restrain lipid dysregulation and ferroptosis

**DOI:** 10.3389/fphar.2026.1852232

**Published:** 2026-08-07

**Authors:** Daoping Wang, Yu Mou, Xuemei Wu, Hongji Wang, Qianjun Wang, Qing Rao, Enming Hu, Qing Li, Xiuyan Zheng, Lei Huang

**Affiliations:** 1 State Key Laboratory of Discovery and Utilization of Functional Components in Traditional Chinese Medicine, Natural Products Research Center of Guizhou Province, Guizhou Medical University, Guiyang, China; 2 School of Pharmaceutical Sciences, Guizhou Medical University, Guiyang, China; 3 Department of Hepatobiliary Surgery, Southwest Hospital, Third Military Medical University (Army Medical University), Chongqing, China; 4 Guizhou Institute of Integrated Agriculture Development, Guiyang, China

**Keywords:** ferroptosis, inflammation, lipid metabolism, metabolic dysfunction-associated steatohepatitis, wogonoside

## Abstract

**Background:**

Metabolic dysfunction-associated steatohepatitis (MASH) is a progressive form of metabolic dysfunction-associated steatotic liver disease with limited pharmacological treatment options. Wogonoside (WOG), a flavonoid isolated from Scutellaria baicalensis, exhibits anti-inflammatory, antioxidant, and lipid-lowering activities; however, its effects and underlying mechanisms in MASH remain unclear.

**Methods:**

A high-fat and high-fructose diet (HFFD)-induced mouse model of MASH and free fatty acid (FFA)-induced HepG2 cell steatosis model were used to evaluate the hepatoprotective effects of WOG. Liver histopathology, serum biochemical parameters, glucose tolerance, lipid accumulation, oxidative stress, ferroptosis-associated indicators, transcriptomic profiling, and expression of relevant proteins were assessed. The anti-ferroptotic effects of WOG were further examined using erastin and ferrostatin-1 in HepG2 cells.

**Results:**

WOG administration ameliorated HFFD-induced obesity, hepatic steatosis, hepatocellular ballooning, liver injury, dyslipidemia, insulin resistance, oxidative stress, and inflammatory responses in mice. Transcriptomic analysis indicated that WOG-responsive genes were enriched in lipid metabolism, peroxisome proliferator-activated receptor signaling, AMPK signaling, and ferroptosis-related pathways. In liver tissues and FFA-treated HepG2 cells, WOG increased AMPK phosphorylation and upregulated the expression of PGC-1α, PPARα, and CPT-1, while reducing the expression of SREBP-1c and its downstream lipogenic proteins, including ACLY, ACACA, FASN, and SCD-1. These changes were accompanied by decreased hepatic and intracellular triglyceride accumulation. In addition, WOG reduced reactive oxygen species, restored mitochondrial membrane potential, increased Nrf2 and HO-1 expression, and attenuated inflammatory responses. WOG also decreased Fe^2+^ accumulation and lipid peroxidation, restored glutathione homeostasis, and increased xCT and GPX4 expression in vivo and in vitro. Moreover, WOG counteracted erastin-induced ferroptosis-related changes and exhibited effects similar to those of ferrostatin-1 in FFA-treated HepG2 cells.

**Conclusion:**

WOG alleviates experimental MASH by improving hepatic lipid metabolic homeostasis and suppressing ferroptosis-associated injury. These protective effects are associated with activation of AMPK signaling and restoration of the xCT/GPX4 antioxidant defense axis. WOG may therefore represent a promising candidate for the pharmacological management of MASH.

## 1 Introduction

Metabolic dysfunction-associated steatotic liver disease (MASLD) ranks among the most prevalent chronic liver diseases worldwide, affecting up to 30.2% of the global population (Younossi et al., 2018; Amini-Salehi et al., 2024). Metabolic dysfunction-associated steatohepatitis (MASH) constitutes a severe and progressive stage of MASLD, pathologically defined by hepatic steatosis, hepatocyte damage (ballooning), and inflammation (Chalasani et al., 2018). The pathogenesis is initiated by excessive lipid accumulation within hepatocytes, which subsequently induces oxidative stress, inflammatory cascades, and programmed cell death, thereby driving the transition from simple steatosis (MASLD) to MASH (Schuster et al., 2018). Epidemiological studies estimate that 20%–30% of individuals with MASLD may progress to MASH (Söderberg et al., 2010), which itself has a global prevalence of approximately 5.27%—a figure that continues to rise annually (Younossi et al., 2023). If left untreated, MASH can advance irreversibly to liver fibrosis, cirrhosis, and hepatocellular carcinoma, posing a substantial threat to public health and imposing significant socioeconomic burdens (Rinella, 2015).

The pathogenesis of MASH is multifactorial, including a complex interplay of insulin resistance, dysregulated lipid metabolism, oxidative stress, lipid peroxidation, and chronic inflammation (Schuster et al., 2018). Chronic consumption of a high-fat and high-sugar diet elevates circulating free fatty acids (FFAs), which in turn activate hepatic lipogenic pathways, promoting *de novo* lipogenesis, leading to lipid accumulation. With exacerbation of lipid droplet accumulation in hepatic, there is a further exacerbation of systemic insulin resistance, oxidative stress, and activation of pro-inflammatory signaling (Tsuchiya et al., 2013), while impairing hepatic fatty acid *β*-oxidation (Liu et al., 2018). AMP-activated protein kinase (AMPK) serves as a central regulator of cellular energy homeostasis and plays an indispensable role in the process of lipid metabolism (Herzig and Shaw, 2018). Under conditions of lipid metabolic dysregulation, AMPK activity is suppressed, resulting in the over-activation of sterol regulatory element-binding protein 1 (SREBP-1), which subsequently upregulates key lipogenic genes (including *Acly*, *Acaca*, *Scd-1*, and *Fasn*), thereby accelerating TG synthesis and hepatic steatosis (Lu et al., 2023). Previous research indicates that the AMPK can also inhibit oxidative stress and lipid peroxidation caused by iron overload in the hepatic by reducing the polyunsaturated fatty acids levels (Wang X. et al., 2023; Zhong et al., 2023). Additionally, AMPK, which is vital for maintaining mitochondrial balance, activation enhances the expression of peroxisome proliferator-activated receptor gamma coactivator 1-alpha (PGC-1α), peroxisome proliferator-activated receptor alpha (PPARα), and carnitine palmitoyltransferase-1 (CPT-1), promoting fatty acid *β*-oxidation, and also inhibiting the expression of lipogenic and inflammatory mediators, collectively attenuating the progression of MASH (Barroso et al., 2011). Therefore, the screening of novel AMPK activators to regulate AMPK-mediated fatty acid oxidation and lipid synthesis and improve lipid metabolism disturbance provides a new strategy for the treatment of MASH. Emerging evidence has identified ferroptosis, an iron-dependent regulated cell death, as playing a critical role in the pathogenesis of MASH (Chen et al., 2022; Zhu et al., 2025). Ferroptosis is characterized by the iron-driven accumulation of lipid peroxides and lipophilic reactive oxygen species, primarily resulting from intracellular iron overload (Dixon et al., 2012). According to previous studies, the xCT/GPX4 axis-mediated inhibition system of ferroptosis is involved in the reduction of lipid peroxides and reactive oxygen species (Sui et al., 2024). Solute carrier family 7 member 11 (SLC7A11/xCT), a cystine/glutamate antiporter, plays a key role in maintaining intracellular glutathione (GSH) levels (Banjac et al., 2008). Subsequently, glutathione peroxidase 4 (GPX4) utilizes GSH to reduce phospholipid hydroperoxides by converting to its oxidized form (GSSG), thereby suppressing the lipid peroxidation chain reactions that drive ferroptosis (Ursini and Maiorino, 2020). Therefore, GSH depletion may be a primary cause of hepatic ferroptosis due to the suppression of GSH synthesis resulting from the inhibition of SLC7A11 expression during the progression of MASH (Banjac et al., 2008; Ursini and Maiorino, 2020). Furthermore, iron overload-mediated production of lipid peroxides can stimulate the hepatic macrophages to release the pro-inflammatory cytokines (TNF-α, IL-6, and IL-β), exacerbating liver inflammation and lipid metabolism disorders, and promoting MASH progression to fibrosis (Yang et al., 2022). Thus, inhibiting hepatic ferroptosis by activating the xCT/GPX4 signaling pathway represents a promising therapeutic strategy for ameliorating MASH.

*Scutellaria baicalensis,* a traditional Chinese herbal medicine with a long history of use, occupies an important position in both the pharmaceutical and food industries due to its rich medicinal value (Zhao et al., 2016). Wogonoside (WOG), a major flavonoid compound derived from *S. baicalensis*, exhibits multiple pharmacological activities, including antioxidant properties (Li et al., 2025), alleviates inflammation (Zhu et al., 2024), and inhibits the progression of fibrosis (Liu et al., 2022). Recent studies indicate that WOG may confer protection against MASLD by suppressing inflammatory responses and the oxidative stress pathway (Jiang et al., 2020). However, its efficacy and underlying mechanisms in the context of MASH remain unclear. To address this issue, we used an HFFD-induced mouse model of MASH. This model was selected because chronic exposure to high dietary fat and fructose reflects major nutritional and metabolic risk factors for human MASH, including obesity, insulin resistance, hepatic lipid accumulation, oxidative stress, and inflammation. Compared with the methionine- and choline-deficient diet model, which induces steatohepatitis but is commonly associated with body weight loss and lacks several key metabolic features of human MASH, the HFFD model provides a more metabolically relevant platform for evaluating interventions targeting lipid metabolic dysfunction and lipotoxicity-related liver injury. Although the AMLN diet also reproduces several metabolic and histological features of MASH, the HFFD model was considered suitable for the present study because it directly reflects excessive fat and fructose intake and reliably induces metabolic stress, steatosis, inflammation, and oxidative damage, all of which are closely associated with ferroptosis-related hepatocellular injury. Therefore, this study aimed to determine whether WOG ameliorates MASH by modulating lipid metabolic reprogramming and suppressing ferroptosis, with particular emphasis on the involvement of AMPK signaling and the xCT/GPX4 antioxidant defense axis.

## 2 Materials and methods

### 2.1 Materials and reagents

Human hepatocarcinoma cell line (HepG2), mouse mononuclear macrophage cell line (Raw264.7), and mouse hepatic macrophage cell line (Kupffer) were purchased from the Chinese Academy of Sciences (Shanghai, China). Dulbecco’s modified Eagle’s medium (DMEM) and RPMI-1640 were obtained from Gibco (Grand Island, NY, USA). Fetal bovine serum (FBS) from North America was obtained from VisTech (VisTech, SE100-B, South America). Wogonoside (WOG) was obtained from Shanghai Aladdin Biochemical Technology Co., Ltd., (Shanghai, China). Free fatty acid (FFA), Bovine Serum Albumin (BSA), and Oil Red O (ORO) were purchased from Sigma-Aldrich (St. Louis, MO, USA). The Reactive Oxygen Species (ROS) Assay Kit and Mitochondrial Membrane Potential Assay Kit with JC-1 were purchased from Solarbio Science and Technology Co., Ltd., (Beijing, China). The Cell Ferrous Iron (Fe^2+^) Assay Kit was purchased from MedChemExpress (New Jersey, USA). Liperfluo was purchased from Dojindo Laboratories (Kumamoto, Japan). The Nitric Oxide (NO) Test Kit was purchased from Beyotime Biotechnology Co., Ltd., (Shanghai, China). Erastin and Ferrostatin-1 (Fer-1) were obtained from GlpBio Technology (Montclair, CA, USA).

### 2.2 Animal experiments

All animal experiments were approved by the Institutional Animal Care and Use Committee of Guizhou Medical University (Approval 2101004). Male C57BL/6J mice (6 weeks old, 20 ± 2 g) were purchased from GemPharmatech Co., Ltd., (Jiangsu, China) and housed under specific pathogen-free (SPF) conditions at 20 °C–25 °C and 60% humidity, with a 12 h light/dark cycle. Mice had free access to food and water throughout the study. After 1 week of acclimatization, all mice were randomly divided into two groups: (1) Control (CN) group, fed a standard chow diet (8 mice); and (2) high-fat and high-fructose diet (HFFD) group (n = 40), fed a diet containing 60% kcal from fat (D12492, Research Diet, New Brunswick, USA) plus 10% fructose. After 14 weeks of continuous HFFD feeding, the HFFD-fed mice were further randomized into five groups (n = 8/group): (1) HFFD group; (2) ATO group, HFFD + atorvastatin (ATO, 10 mg/kg; positive control); (3) WOG-L group, HFFD + low-dose WOG (2.5 mg/kg); (4) WOG-M group, HFFD + medium-dose WOG (5 mg/kg); (5) WOG-H group, HFFD + high-dose WOG (10 mg/kg). Treatments or vehicle (saline) were administered daily by oral gavage for 12 weeks. The dose range of WOG was selected based on previous *in vivo* studies reporting pharmacological effects of WOG at similar or higher doses, together with preliminary tolerability observations in our experimental setting (Li et al., 2025; Zhu et al., 2024; Liu et al., 2022). During the treatment period, no obvious adverse effects, abnormal behavior, or treatment-related mortality were observed in WOG-treated mice. Body weight was measured weekly. At the end of the experiment, fasting blood glucose (FBG) was measured, and an intraperitoneal glucose tolerance test (IPGTT) was performed.

### 2.3 Histological analysis

At the end of the experiment, mice were euthanized by gradual exposure to carbon dioxide (CO_2_) at a flow rate of 40% chamber volume per minute, in accordance with the AVMA Guidelines for the Euthanasia of Animals (2020 edition). After euthanasia, liver tissues were collected, weighed, and recorded. A portion of the liver tissue was fixed with 4% paraformaldehyde solution, dehydrated, embedded in paraffin, and sectioned. The sections were stained with H&E, ORO, and immunohistochemistry (IHC) according to previously established protocols (Mou et al., 2025). Stained sections were imaged under a microscope to obtain the liver tissue staining results.

### 2.4 Serum and liver biochemical analysis

Mice were euthanized using carbon dioxide inhalation, and whole blood was collected via apical puncture. The serum was collected by centrifuging at 3,000 rpm for 15 min at 4 °C. Levels of TG, TC, LDL-C, HDL-C, AST, ALT, MDA, and GSH-Px in the serum of mice were determined using commercial assay kits (Nanjingjiancheng, Nanjing, China). Serum levels of TNF-α, IL-6, IL-1β, and INS in serum were measured using ELISA kits (Nanjingjiancheng, Nanjing, China) according to the instructions. HOMA-IR was calculated as follows:

HOMA−IR=FBG×FINS/22.5.

### 2.5 Liver transcriptome sequencing

Total RNA was extracted from frozen liver tissues (−80 °C) using Trizol reagent. RNA concentration was measured using a NanoDrop 2000 spectrophotometer. RNA quality control and sequencing were performed by Wuhan Huada Technology Co., Ltd. Raw sequencing data were filtered using SOAPnuke, followed by data analysis and visualization using the Dr. Tom data mining system. Differentially expressed genes (DEGs) were identified based on the criteria of |log_2_FC| > 1 and P < 0.05. Gene Ontology (GO) and Kyoto Encyclopedia of Genes and Genomes (KEGG) enrichment analyses were performed on the DEGs using Phyper. Finally, the results were visualized using TBtool software.

### 2.6 Detection of Fe^2+^ content in liver tissue

Fresh liver tissue (0.1 g) from each group was homogenized, and Fe^2+^ content was measured using a commercial iron assay kit according to the manufacturer’s instructions (Elabscience Biotechnology Co., Ltd., Wuhan, China).

### 2.7 Cell cultures and cytotoxicity assay

HepG2 cells were cultured in DMEM medium containing 10% FBS. All cells were kept at 37 °C in a 5% CO_2_ atmosphere. For cytotoxicity assessment, cells in the logarithmic growth phase were seeded into 96-well plates at 1 × 10^4^ cells/well. After attachment, cells were treated with WOG (2.5, 5, or 10 μM) for 48 h. Then, 10 μL of MTT solution (5 mg/mL) was added to each well and incubated for 4 h. The formazan crystals were dissolved in 160 μL DMSO, and absorbance was measured at 490 nm using a microplate reader. Cell viability was expressed as a percentage of the control group.

### 2.8 Lipid analysis in HepG2 cells

HepG2 cells were seeded in 6-well plates at 1.5 × 10^5^ cells/well during the logarithmic growth phase. After attachment, the cells were treated with 0.3 mM oleic acid/palmitic acid (OA: PA = 2:1) for 24 h to induce steatosis. Remove the cell supernatant and add fresh DMEM medium containing WOG (2.5 μM, 5 μM, and 10 μM) or ATO (10 μM) to treat the cells for 48 h. Intracellular TG content was quantified using a commercial TG assay kit according to the manufacturer’s instructions. Lipid accumulation was also assessed by ORO staining as previously described (Huang et al., 2024).

### 2.9 Detection of ROS, mitochondrial membrane potential, lipid peroxides, and Fe^2+^ levels in HepG2 cells

HepG2 cells were incubated with the following fluorescent probes: DCFH-DA for reactive oxygen species (ROS), JC-1 for mitochondrial membrane potential (ΔΨm), Liperfluo for lipid peroxides, and RhoNox-1 for Fe^2+^. After 20 min of incubation at 37 °C in the dark, cells were washed with PBS. Fluorescence was visualized using an inverted fluorescence microscope. For quantitative analysis, cells were trypsinized, resuspended in PBS, and analyzed by flow cytometry.

### 2.10 Western blot analysis

Treated cells or liver tissues were lysed in RIPA buffer containing 1% PMSF and 1% phosphatase inhibitor, which was then homogenized and incubated on ice for 40 min. Lysates were centrifuged at 12,000 rpm for 15 min at 4 °C, and the supernatant was collected. Protein concentration was determined using a BCA assay. Proteins (50 μg per lane) were separated by SDS-PAGE and transferred to PVDF membranes (0.22 μm). After blocking with 5% skim milk or 3% BSA for 2 h, tmembranes were incubated overnight at 4 °C with the following primary antibodies: β-actin (AF7018, Affinity), CPT-1 (ab234111, Abcam), PPARα (ab178865, Abcam), AMPKα (AF6423, Affinity), p-AMPK (AF3423, Affinity), Acaca (ab45174, Abcam), SCD-1 (#2438, CST), SREBP-1c (AF6283, Affinity), Fasn (DF6106, Affinity), Acly (AF7832, Affinity), PGC-1α (AF5395, Affinity), xCT (DF12509, Affinity), GPX4 (DF6701, Affinity), Nrf2 (AF0639, Affinity), and HO-1 (AF5393, Affinity). After primary antibody incubation, wash three times with TBST, then incubate with secondary antibody for 1 h. Finally, Protein bands were visualized using an Odyssey imaging system.

### 2.11 Statistical analysis

All experimental data were statistically analyzed using SPSS 26.0 software (IBM SPSS, Chicago, IL, USA) and are presented as mean ± standard error. Data were analyzed using T-tests and one-way ANOVA, and *P* < 0.05 was considered statistically significant. The results were graphed using GraphPad Prism 8.0.2 software.

## 3 Results

### 3.1 WOG effectively alleviates MASH in mice caused by HFFD

**FIGURE 1:**
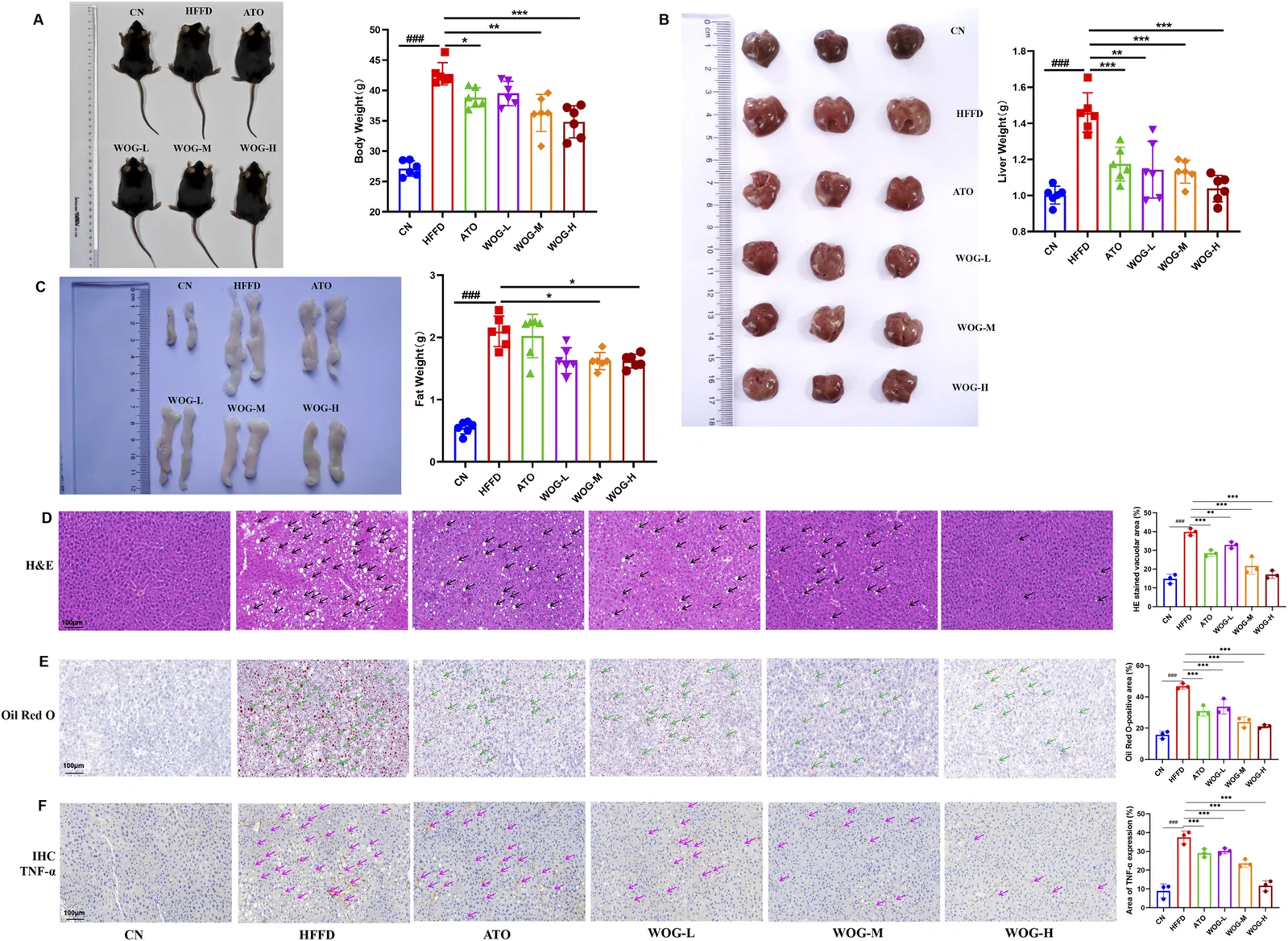
Effect of Wogonoside (WOG) on HFFD-induced MASH mice. **(A–C)** Changes in body weight, liver, and adipose tissue were observed in MASH mice treated with WOG; Data are expressed as mean ± standard deviation (n = 6). **(D)** Liver tissue stained with H&E (magnification ×200) and **(E)** Oil red O (magnification ×200) shows hepatic lipid accumulation (steatosis) in the MASH mice. **(F)** Representative images of immunohistochemistry staining (IHC) of TNF-α (magnification ×200); Experimental data have been expressed as mean ± standard deviation (n = 3). ^###^P < 0.001 compared with the CN group; *P < 0.05, **P < 0.01, and ***P < 0.001 compared with the HFFD group. Note: Black arrows denote fat vacuoles, green arrows indicate lipid droplets, and purple arrows signify TNF-α positivity. In the IHC experiment results, cell nuclei appear blue, and DAB-stained positive expression appears brownish-yellow.

To investigate the therapeutic potential of Wogonoside (WOG), we employed a mouse model of MASH induced by a high-fat and high-sugar diet (HFFD). Compared to the control (CN) group, HFFD-induced mice developed severe obesity, hepatomegaly, and increased abdominal fat mass, with increases of 55.4%, 48.0%, and 281.8%, respectively (Figures 1A–C). Following intervention with WOG, those changes were significantly reversed in a dose-dependent manner, with the effect of the lower-dose WOG (2.5 mg/kg) being comparable to that of the positive control atorvastatin (ATO). Meanwhile, histological analysis further confirmed the protective role of WOG. As shown in Figure 1D, H&E staining analysis exhibited that WOG alleviated hepatic steatosis, reduced hepatocyte ballooning, and decreased lipid vacuole size compared with the HFFD group. Consistent with the results of H&E staining, ORO staining revealed a dose-dependent reduction in hepatic lipid droplets following WOG treatment (Figure 1E). Furthermore, we examined the changes in expression of TNF-α, which is an important indicator of MASH, in HFFD-induced MASH mice through IHC experiments. Results displayed that the TNF-α expression level in MASH mice was markedly reduced after treatment with WOG compared to the HFFD group, suggesting that WOG inhibited the inflammatory response in the liver of mice with MASH induced by HFFD (Figure 1F). According to the results above, we also found that the livers of mice treated with a high dose of WOG (10 mg/kg) recovered and were comparable to those of normal mice, which was superior to the positive control group ATO (10 mg/kg). These results indicate that WOG can effectively alleviate lipid accumulation, hepatic steatosis, and inflammation infiltration in MASH mice caused by HFFD.

### 3.2 WOG improves serum biochemistry indicators in HFFD-induced MASH mice

**FIGURE 2:**
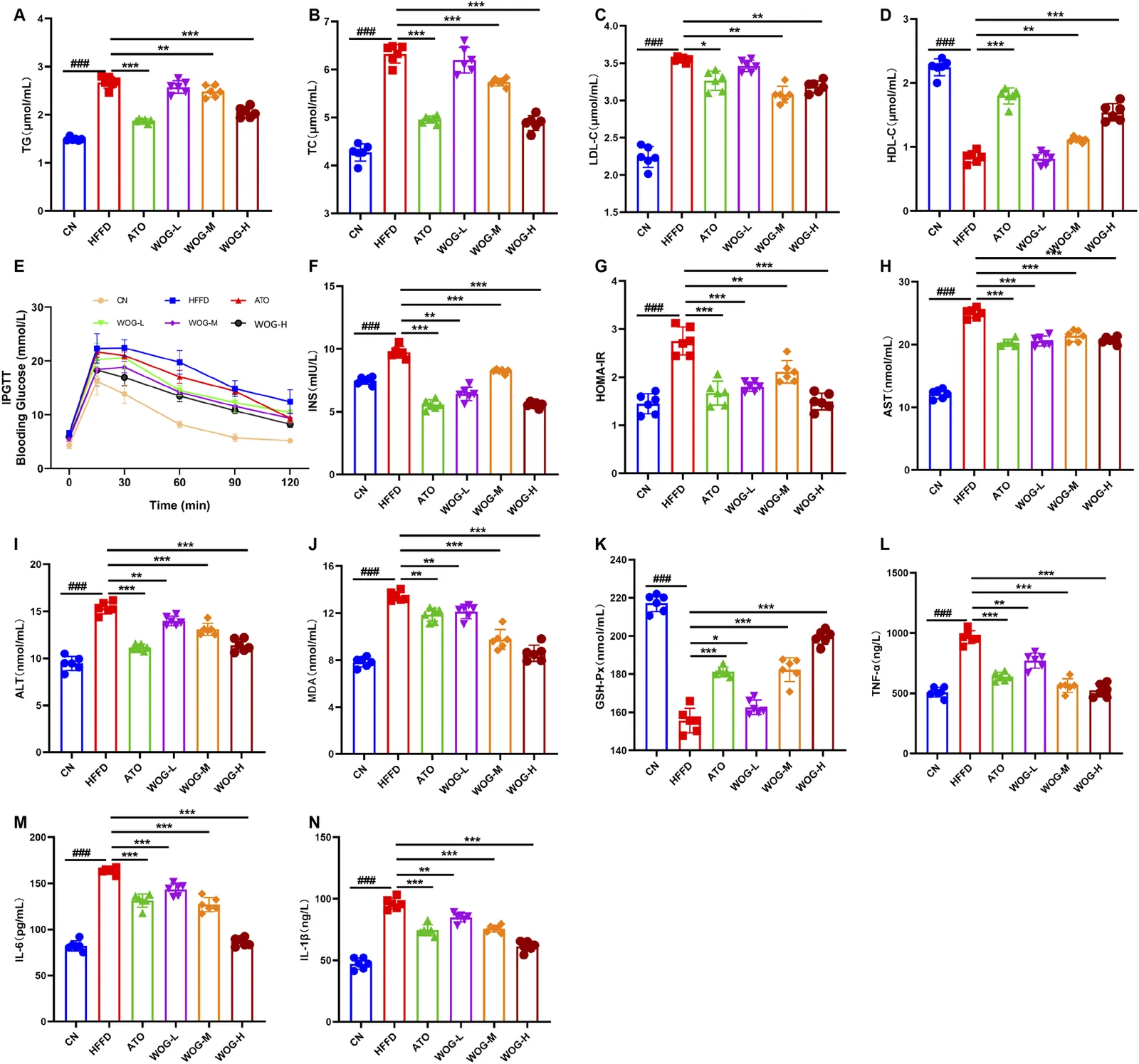
Effects of WOG on serum biochemistry indicators in HFFD-induced MASH mice. **(A–D)** The detection of serum TC, TG, LDL-C, and HDL-C levels in MASH mice. **(E)** IPGTT. **(F)** Serum INS levels in MASH mice. **(G)** HOMA-IR. **(H–N)** The levels of serum AST, ALT, MDA, GSH-Px, TNF-α, IL-6, and IL-1β were measured. Experimental data have been expressed as mean ± standard deviation (n = 6); ^###^P < 0.001 compared with the CN group; *P < 0.05, **P < 0.01, and ***P < 0.001 compared with the HFFD group.

To further investigate the alleviating effect of WOG on MASH mice, we examined the serum biochemistry indicators. Results exhibited that the serum lipid profile balance of mice with MASH was disrupted by long-term feeding of the HFFD diet. Specifically, the serum levels of TG, TC, and LDL-C in the HFFD group increased by 79.1%, 48.2%, and 58.3%, respectively, while the HDL-C level was decreased by 62.5% compared to the CN group (Figures 2A–D). Following treatment with WOG, TG, TC, and LDL-C levels in MASH mice showed a gradual downward trend with increasing WOG dose, while HDL-C displayed an opposite change. In addition, the HFFD diet also led to a decrease in glucose tolerance and insulin sensitivity of the MASH mice. As shown in Figure 2E, WOG administration dose-dependently alleviated the glucose intolerance, especially at a high dose of WOG, which was comparable to the positive control group (ATO). Moreover, compared with the HFFD group, the WOG intervention groups exhibited a significant reduction in serum insulin levels, with the WOG-H group demonstrating a 43.4% decrease (Figure 2F). And the HOMA-IR index further confirmed that the insulin sensitivity was markedly elevated after WOG treatment, as presented in Figure 2G. Meanwhile, ATO (10 mg/kg) intervention also restored the lipid profile balance and enhanced glucose tolerance and insulin sensitivity in HFFD-induced MASH mice; however, its overall effect was significantly lower than that of WOG.

Next, HFFD feeding also induced hepatic damage and oxidative stress. Compared to the CN group, mice in the HFFD group exhibited significant increases in serum levels of AST (1.1-fold), ALT (0.6-fold), and MDA (0.7-fold), alongside a 0.3-fold reduction in GSH-Px. WOG intervention effectively reversed these alterations, dose-dependently lowering AST, ALT, and MDA levels while elevating GSH-Px activity, with the most pronounced effects observed in the WOG-H group (Figures 2H–K). Concurrently, serum levels of the pro-inflammatory cytokines TNF-α, IL-6, and IL-1β in MASH mice were increased by 90.0%, 99.1%, and 103.7%, respectively, in the HFFD group, and these increases were dose-dependently suppressed by WOG (Figures 2L–N). Notably, the anti-inflammation activity of WOG was also demonstrated in the LPS-induced RAW264.7 and Kupffer cells by examining the expression of inflammatory cytokine genes (*TNF-α*, *IL-6*, and *IL-1β*) (Supplementary Figure S2). These data suggest that WOG can alleviate liver damage, oxidative stress, and inflammatory responses in MASH mice caused by lipotoxicity following long-term feeding of a HFFD diet, and affirm its superior hepatoprotective advantage over conventional ATO therapy.

### 3.3 Liver transcriptomics analysis

**FIGURE 3:**
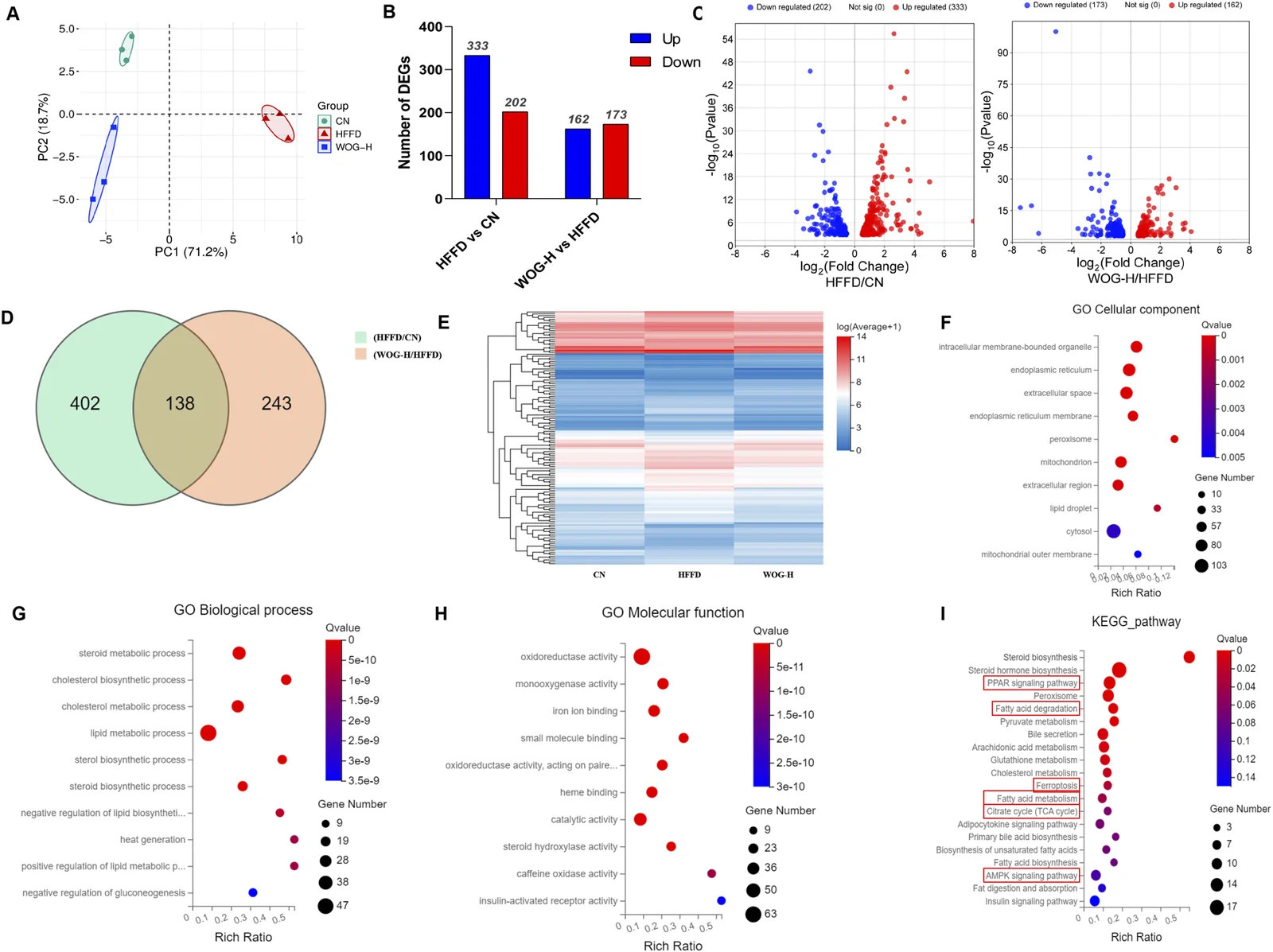
Transcriptomic analysis of WOG treatment. **(A)** PCA. **(B)** Screening differentially expressed genes by sequencing results of the whole transcriptome. **(C)** Volcano map of differentially expressed genes. **(D)** Venn diagram of differentially expressed genes. **(E)** Heatmap of differentially expressed genes clustering. **(F–H)** GO functional enrichment analysis of the relevant biological processes, cellular components, and molecular functions of interacting genes of differentially expressed genes. **(I)** KEGG pathway analysis was performed as pathway annotation.

To gain mechanistic insight, we performed RNA sequencing on liver tissues from the CN, HFFD, and WOG-H groups. As shown in Figure 3A, principal component analysis (PCA) showed a clear separation among the three groups, with the WOG-H group clustering closer to the CN group than to the HFFD group, indicating that WOG treatment shifts the hepatic transcriptome towards a healthier state. A total of 540 differentially expressed genes (DEGs) were identified between the CN and HFFD groups (333 up, 202 down) and 381 DEGs between the HFFD and WOG-H groups (162 up, 173 down), based on the criteria of |log_2_FC| > 1 and P < 0.05 (Figures 3B,C). An intersection analysis identified 129 core genes whose expression was altered by HFFD and subsequently normalized by WOG (Figures 3D,E), suggesting that WOG largely reversed the dysregulation of genes involved in lipid metabolism induced by HFFD. Furthermore, GO enrichment analysis of DEGs between the HFFD and WOG-H groups associated these genes with cellular components, including the endoplasmic reticulum, peroxisome, mitochondrion, and lipid droplet (Figure 3F). Biological processes were enriched for cholesterol biosynthesis, lipid metabolism, and negative regulation of lipid synthesis (Figure 3G), while molecular functions included oxidoreductase activity, iron ion binding, and insulin-activated receptor activity (Figure 3H). Notably, KEGG pathway analysis suggested that the anti-MASH effects of WOG may be associated with modulating the PPAR signaling pathway, ferroptosis, fatty acid metabolism, and the AMPK signaling pathway (Figure 3I), all of which are closely related to the progression of MASH (Hu et al., 2023; Steinberg and Hardie, 2023).

### 3.4 WOG restores hepatic lipid homeostasis by regulating the AMPK signaling pathway

**FIGURE 4:**
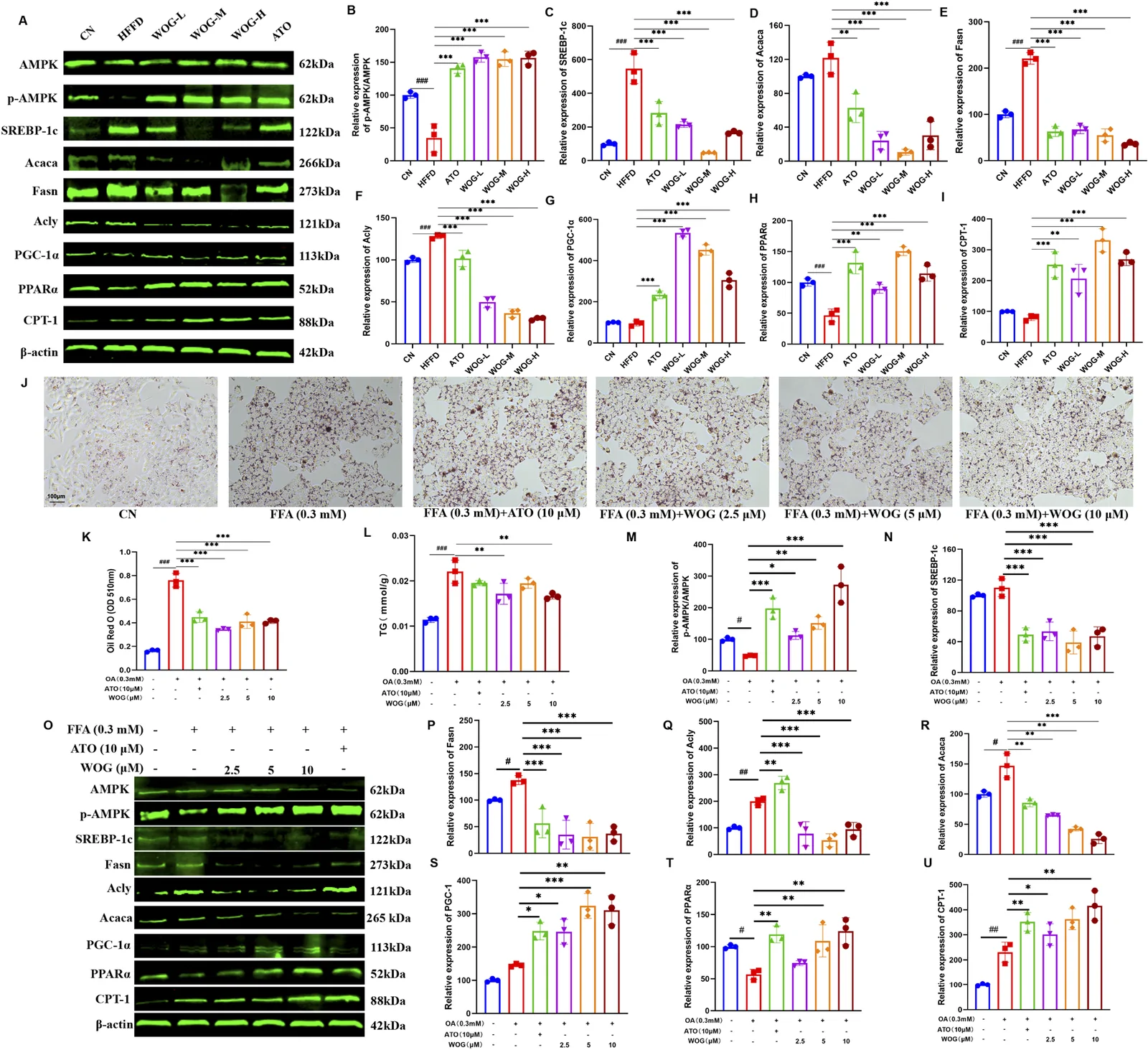
Effect of WOG on lipid metabolism disorder in HFFD-induced MASH mice and FFA-induced HepG2 cells. **(A–I)** Western blot was used to detect the expression of lipid metabolism-related proteins, including AMPK, p-AMPK, SREBP-1c, Acaca, Fasn, Acly, PGC-1α, PPARα, and CPT-1 in the liver of HFFD-induced MASH mice. **(J,K)** ORO staining analysis reveals the lipid accumulation in FFA-induced HepG2 cells. **(L)** The detection of TG levels in FFA-induced HepG2 cells. **(M–U)** Western blot detection of AMPK, p-AMPK, SREBP-1c, Fasn, Acly, Acaca, PGC-1α, PPARα, and CPT-1 expression in FFA-induced HepG2 cells. Experimental data have been expressed as mean ± standard deviation (n = 3); ^#^P < 0.05, ^##^P < 0.01, and ^###^P < 0.001 compared with the CN group; *P < 0.05, **P < 0.01, and ***P < 0.001 compared with the HFFD or FFA group.

Liver transcriptomics and serum lipid profile both demonstrated that WOG alleviates HFFD-induced MASH was correlated with regulating lipid metabolism. To investigate the molecular pathways underlying, we examined the lipid metabolism-related proteins in the liver tissue of MASH mice by Western blot. Compared to the CN group, mice in the HFFD group exhibited significant increases in liver proteins levels of SREBP-1c (4.5-fold), Fasn (0.13-fold), Acaca (1.2-fold), Acly (0.27-fold), which are key lipogenic proteins in the SREBP-1c pathway, and along with a 0.65-fold reduction in p-AMPK/AMPK. Meanwhile, HFFD feeding also resulted in lower levels of PGC-1α, PPARα, and CPT-1 proteins, which are involved in fatty acid oxidation through the PGC-1α/PPARα pathway, by 10%, 53.3%, and 23.5%, respectively, compared to the CN group. WOG intervention effectively reversed these expression patterns, suppressing lipogenic genes and promoting fatty acid oxidation (Figures 4A–I). Notably, the efficacy of WOG surpassed that of atorvastatin.

Furthermore, we employed a cellular model of lipid accumulation induced by free fatty acids (FFA) in HepG2 cells to confirm the effect of WOG on lipid metabolism *in vitro*. As shown in Figures 4J,K, ORO staining confirmed that WOG significantly reduced intracellular lipid droplet accumulation. Meanwhile, FFA stimulation increased cellular TG content by 82.4%, while WOG treatment reduced TG content in a concentration-dependent manner, demonstrating a superior effect compared to ATO (Figure 4L). Mirroring the *in vivo* findings, WOG downregulated the protein levels of SREBP-1c, Acly, and Fasn, and upregulated PGC-1α, PPARα, CPT-1, and p-AMPK in FFA-treated HepG2 cells (Figures 4M–U). These results demonstrate that WOG alleviates hepatic lipid accumulation by activating AMPK to inhibit the SREBP-1c pathway and activate the PGC-1α/PPARα pathway, thereby decreasing the lipid droplet deposition in hepatic cells.

### 3.5 WOG exerts its protective effects by inhibiting oxidative stress

**FIGURE 5:**
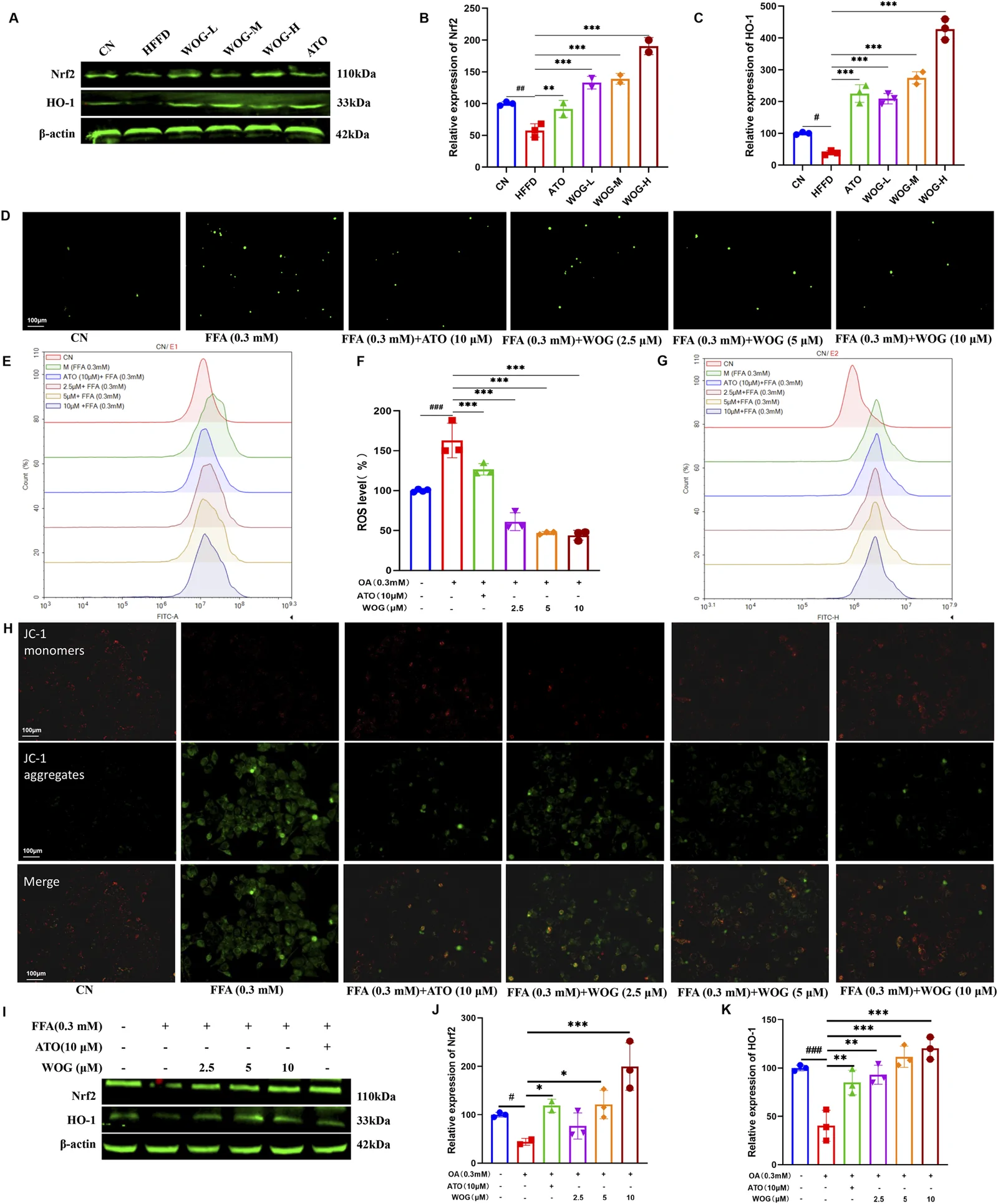
WOG inhibits oxidative stress and cellular damage. **(A–C)** Western blot detection of Nrf2 and HO-1 protein expression in liver tissue of MASH mice. **(D–F)** The ROS levels in FFA-induced HepG2 cells were measured. **(G,H)** Mitochondrial activity detected by JC-1 staining in FFA-induced HepG2 cells. **(I–K)** Western blot detection of Nrf2 and HO-1 protein expression in FFA-induced HepG2 cells. Experimental data have been expressed as mean ± standard deviation (n = 3); ^#^P < 0.05 and ^###^P < 0.001 compared with the CN group; *P < 0.05, **P < 0.01, and ***P < 0.001 compared with the HFFD or FFA group.

MASH development is closely associated with oxidative stress resulting from lipotoxicity, and the ongoing presence of oxidative stress further promotes the progression of the disease. In the present study, transcriptomic analysis and liver serum biochemistry indicators both confirmed that oxidative stress (Xiong et al., 2022). To further investigate the effect of WOG on the oxidative stress in the liver of MASH mice, we conducted a Western blot analysis of the expression of oxidative stress-related proteins. As depicted in Figures 5A–C, the expression levels of Nrf2 and HO-1 in the HFFD group were significantly downregulated, respectively, compared to the CN group. Following WOG intervention, the expression levels of both Nrf2 and HO-1 were significantly upregulated.

Next, we further investigated the protective effects of WOG through *in vitro* experiments. In FFA-stimulated HepG2 cells, intracellular reactive oxygen species (ROS) levels increased by 63.0%, an effect that was concentration-dependently mitigated by WOG (Figures 5D–F). Using the JC-1 probe, we found that FFA stimulation dissipated the mitochondrial membrane potential (indicated by a shift from red to green fluorescence), which was restored by WOG treatment (Figures 5G,H). Furthermore, WOG upregulated the expression of Nrf2 and HO-1 in these cells, which is consistent with *in vivo* results (Figures 5I–K). These data collectively indicate that WOG inhibits oxidative stress in the context of MASH.

### 3.6 Regulation of WOG on the hepatic ferroptosis in MASH

**FIGURE 6:**
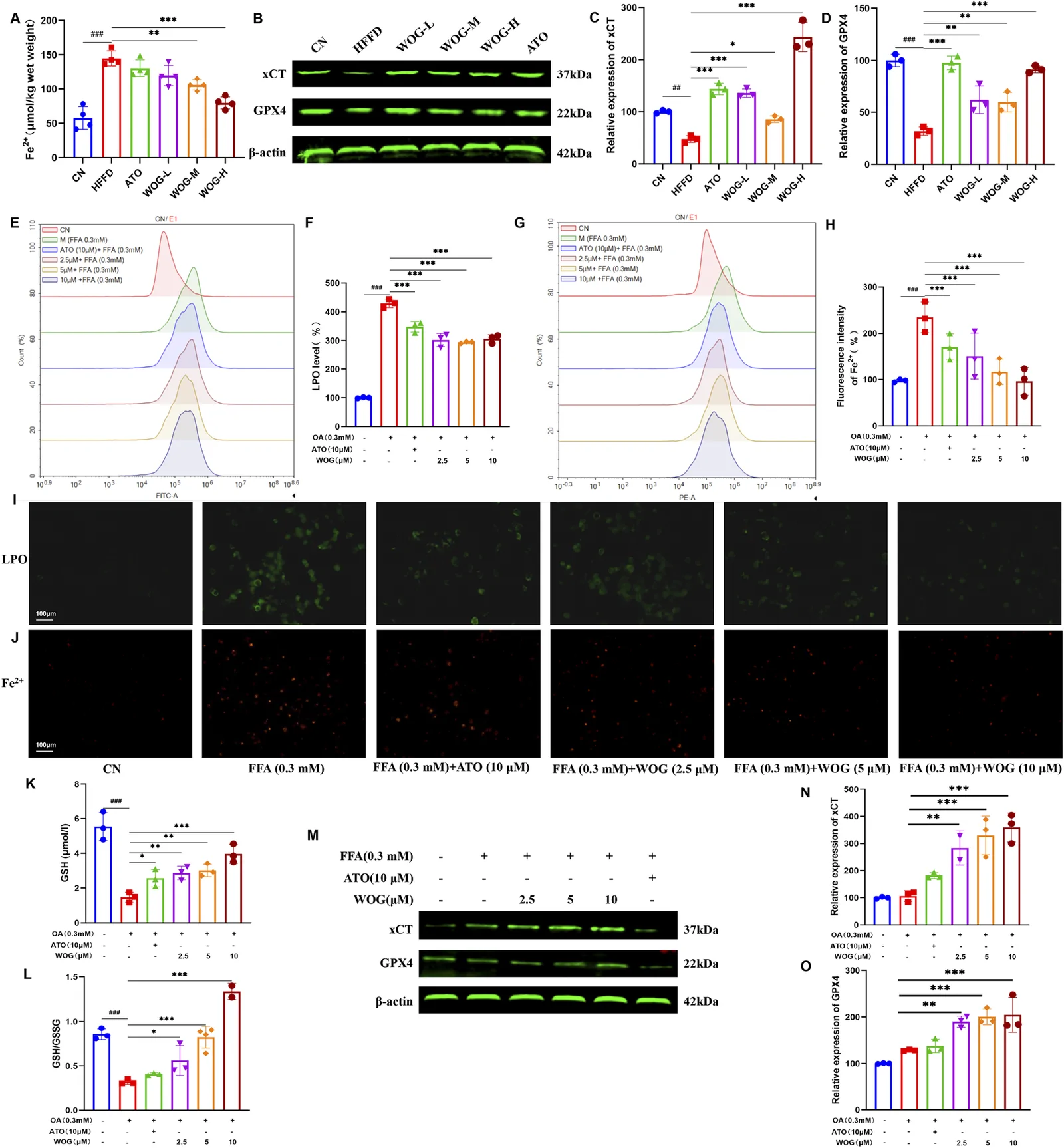
WOG inhibits ferroptosis by modulating the xCT/GPX4 axis. **(A)** Fe^2+^ content in the livers of MASH mice. **(B–D)** Western blot was used to detect the expression of ferroptosis-related proteins, including xCT and GPX4, in the liver of HFFD-induced MASH mice. **(E–J)** The levels of LPO and Fe^2+^ in FFA-induced HepG2 cells were detected by flow cytometry and fluorescence microscopy (magnification ×200). **(K,L)** The detection of GSH and GSH/GSSG in FFA-induced HepG2 cells. **(M–O)** Western blot detection of xCT and GPX4 protein expression. Experimental data have been expressed as mean ± standard deviation (n = 3); ^#^P < 0.05, ^##^P < 0.01, and ^###^P < 0.001 compared with the CN group; *P < 0.05, **P < 0.01, and ***P < 0.001 compared with the HFFD or FFA group.

Our transcriptomic analysis suggested WOG may improve the progression of MASH through a mechanism related to ferroptosis. To confirm the regulatory effect of WOG on ferroptosis, we examined the Fe^2+^ levels and related protein expression. Indeed, hepatic Fe^2+^ levels, a key feature of ferroptosis, were increased by 1.52-fold in HFFD mice compared to CN mice. WOG treatment dose-dependently reduced hepatic Fe^2+^ levels, with a 45.0% decrease in the WOG-H group (Figure 6A). Western blot analysis showed that HFFD feeding downregulated the anti-ferroptotic proteins xCT and GPX4. WOG intervention reversed these changes, markedly upregulating xCT and GPX4 levels (Figures 6B–D).

**FIGURE 7:**
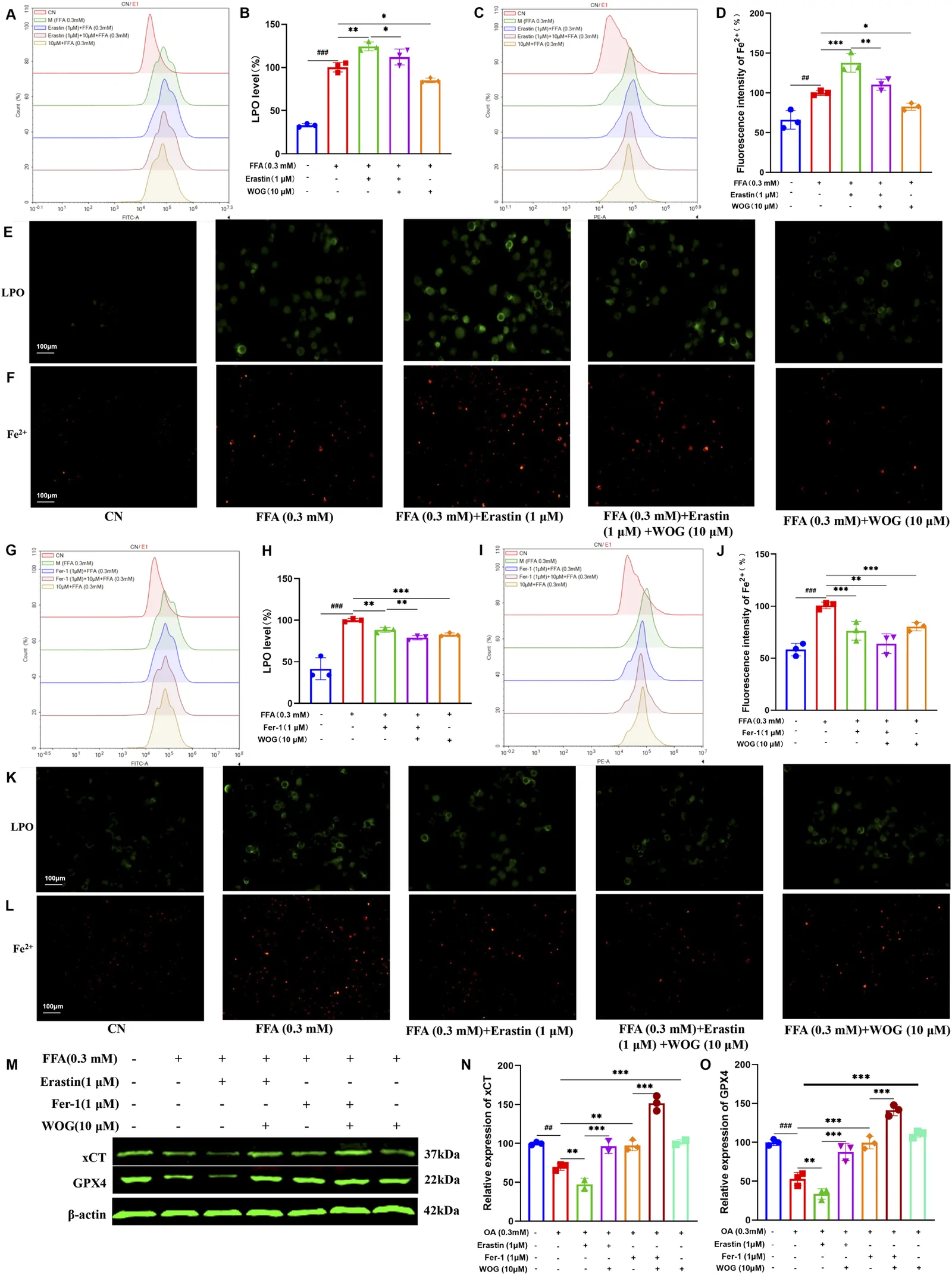
Validating the effect of WOG on the inhibition of ferroptosis induced by the ferroptosis inducer Erastin and the inhibitor Fer-1. **(A–H)** Cellular LPO and Fe^2+^ levels are measured using flow cytometry and fluorescence microscopy (magnification ×200) after WOG and erastin treatment. **(G–L)** The effect of WOG and Fer-1 on LPO and Fe^2+^ levels in FFA-induced HepG2 cells. **(M–O)** Changes in protein (xCT and GPX4) expression levels were detected by Western blot. Experimental data have been expressed as mean ± standard deviation (n = 3); ^##^P < 0.01 and ^###^P < 0.001 compared with the CN group; *P < 0.05, **P < 0.01, and ***P < 0.001 compared with the FFA group.

We further validated the anti-ferroptotic effect *in vitro* using FFA-induced HepG2 cells. FFA stimulation in HepG2 cells led to significant accumulation of lipid peroxides (LPO) and Fe^2+^, as detected by flow cytometry and fluorescence microscopy. WOG treatment concentration-dependently reduced both LPO and Fe^2+^ levels in FFA-induced HepG2 cells (Figures 6E–J). Ferroptosis is critically regulated by the xCT/GPX4 axis. FFA stimulation depleted intracellular glutathione (GSH) and lowered the GSH/GSSG ratio, effects that were reversed by WOG (Figures 6K,L). Consistent with the *in vivo* data, WOG also upregulated xCT and GPX4 protein expression in cells (Figures 6M–O). In addition, we used the ferroptosis inducer Erastin and inhibitor Ferrostatin-1 (Fer-1) to further solidify the effect of WOG on ferroptosis. Results showed that erastin treatment robustly increased cellular LPO and Fe^2+^ levels, and WOG effectively counteracted this effect (Figures 7A–F). Conversely, Fer-1 itself inhibited FFA-induced LPO and Fe^2+^ accumulation, and the addition of WOG produced a similar, non-additive effect (Figures 7G–L). At the protein level, WOG reversed the Erastin-induced downregulation of xCT and GPX4. Similarly, in the presence of FFA, WOG further enhanced the protective protein expression pattern elicited by Fer-1 (Figures 7M–O). These data suggest that WOG can inhibit ferroptosis in hepatocytes by regulating xCT/GPX4 signaling pathway.

## 4 Discussion

MASH represents a progressive stage of MASLD affecting 20%–30% of MASLD patients and is a key driver of cirrhosis and hepatocellular carcinoma (Schuppan et al., 2018; Zhang and Yang, 2021). Lipid metabolism disorder, inflammation, and oxidative stress are core pathogenic features (Lee et al., 2023), yet effective multi-target therapies remain lacking (Xu et al., 2022). Recently, the role of medication and food homology in Chinese herbal medicine for health has attracted significant attention, particularly regarding the advantages of medicine and food homology-derived ingredients in the treatment of chronic diseases (Hou and Jiang, 2013; Yan et al., 2020). Wogonoside (WOG), a flavonoid derived from *S. baicalensis*, possesses anti-inflammatory, antioxidant, and lipid-lowering activitese (Yao et al., 2017; Yu et al., 2020). While WOG has shown potential in ameliorating MASLD (Jiang et al., 2020), its therapeutic role in MASH and the underlying molecular mechanisms have not been fully clarified. In the present study, we demonstrate that WOG markedly alleviates HFFD-induced MASH in mice and FFA-induced hepatocellular lipotoxicity *in vitro*. Mechanistically, our findings indicate that WOG exerts a dual protective effect by restoring hepatic lipid metabolic homeostasis through activation of the AMPK pathway and suppressing ferroptosis through modulation of the xCT/GPX4 axis. These results suggest that WOG may ameliorate MASH by coordinately targeting both the metabolic drivers and ferroptotic consequences of hepatic lipotoxicity.

Hepatic lipid metabolic dysregulation is a central pathological event in MASH progression (Ipsen et al., 2018; Liu et al., 2016; Geng et al., 2021). In this study, WOG administration markedly ameliorated HFFD-induced increases in body weight, hepatic steatosis, hepatocellular ballooning, and massive accumulation of lipid droplets. These histopathological improvements were accompanied by restoration of the serum lipid profile, as evidenced by reduced TG, TC, and LDL-C levels and elevated HDL-C levels, as well as attenuation of liver injury markers, including ALT, AST, and MDA, which is consistent with previous findings (Jiang et al., 2020). In addition, improved glucose homeostasis and insulin sensitivity. Collectively, these findings indicate that WOG exerts broad hepatoprotective effects against HFFD-induced metabolic injury. ATO was included as a positive control in the animal experiments because of its established beneficial effects on dyslipidemia, hepatic steatosis, inflammation, and liver injury in experimental MAFLD/MASH models. Its inclusion was intended to provide a clinically relevant reference for comparison of overall hepatoprotective efficacy, rather than to imply a mechanism identical to that of WOG. While ATO primarily acts through HMG-CoA reductase inhibition and lipid-lowering effects, WOG in the present study appears to protect against MASH mainly through AMPK activation and inhibition of xCT/GPX4-mediated ferroptosis. Transcriptomic analysis further supported the metabolic regulatory effects of WOG. The hepatic transcriptomic profile of WOG-treated mice shifted toward that of the control group, and enrichment analyses showed that the differentially expressed genes were closely associated with lipid metabolism, fatty acid metabolism, PPAR signaling, AMPK signaling, and ferroptosis, which are closely related to lipid metabolism (Hu et al., 2023; Steinberg and Hardie, 2023). These findings provided an unbiased molecular basis for subsequent mechanistic validation and suggested that WOG may exert its protective effects through coordinated regulation of metabolic dysfunction and ferroptosis-related pathways.

Following the transcriptomic evidence implicating lipid metabolic pathways and AMPK signaling in the response to WOG, we further validated key proteins involved in lipid synthesis and fatty acid *β*-oxidation. Western blot analysis indicated that WOG reduced the expression of lipid synthesis-related proteins, including SREBP-1c, Acly, Acaca, Fasn, and SCD-1, while increasing the expression of fatty acid β-oxidation-related proteins, including PGC-1α, PPARα, and CPT-1. SREBP-1c is a key transcription factor in the *de novo* lipogenesis pathway and promotes lipid synthesis by upregulating lipogenic enzymes such as Acly, Acaca, Fasn, and SCD-1 (Bertolio et al., 2019). Conversely, increased PGC-1α expression has been shown to enhance PPARα and CPT-1 expression, thereby accelerating fatty acid *β*-oxidation (Dong et al., 2024; Liao et al., 2024). Notably, WOG increased the phosphorylation level of AMPK, a central energy sensor that suppresses lipid synthesis by inhibiting SREBP-1c expression, while promoting fatty acid *β*-oxidation through the PGC-1α-related pathway (Kahn et al., 2005; Strzyz, 2020; Lan et al., 2021). Additionally, similar regulatory effects were also observed in FFA-induced hepatocyte steatosis in HepG2 cells. Together, these findings suggested that WOG can restore lipid metabolism balance and reduce hepatic lipid accumulation in MASH, at least in part, by activating the AMPK signaling pathway.

Consistent with this AMPK-associated improvement in lipid metabolic balance, WOG also alleviated oxidative stress and inflammatory responses, two pathological processes closely linked to lipid overload and mitochondrial dysfunction in MASH. Inflammation and oxidative stress are critical drivers of MASH progression from simple steatosis to steatohepatitis (Huby and Gautier, 2022). Numerous studies have demonstrated that inflammatory activation in MASH is closely associated with excessive reactive oxygen species (ROS), which can arise from lipid metabolism disorders and impaired mitochondrial function (Rochette et al., 2022). Persistent ROS accumulation may further compromise the Nrf2/HO-1 antioxidant defense pathway, aggravating oxidative stress and hepatocellular inflammatory injury (O'Rourke et al., 2024). In the present study, HFFD feeding significantly decreased the expression of Nrf2 and HO-1 proteins and increased the pro-inflammatory cytokine levels (TNF-α, IL-6, and IL-1β) in MASH mice. Consistently, lipid droplet deposition caused by FFA significantly elevated the ROS levels, disrupted mitochondrial membrane potentials, and reduced Nrf2 and HO-1 protein expression in HepG2 cells. These abnormalities were substantially reversed following WOG administration. These results indicate that WOG reduces the ROS accumulation and suppresses lipotoxicity-induced inflammatory responses, thereby contributing to the attenuation of MASH progression. Given that ROS accumulation and lipid peroxidation are key biochemical events linking oxidative stress to ferroptotic cell death, we next examined whether ferroptosis was involved in the hepatoprotective effects of WOG. Consistent with this possibility, a salient finding of our transcriptomic analysis was the enrichment of the ferroptosis pathway. Ferroptosis is an iron-dependent form of regulated cell death driven by lipid peroxidation and has been increasingly recognized as an important contributor to the progression from simple MASLD to MASH (Kajarabille and Latunde-Dada, 2019; Li et al., 2020; He et al., 2023). Previous studies have shown that hepatic iron overload can promote ROS production, lipid peroxide accumulation, mitochondrial damage, oxidative stress, and inflammatory responses, all of which contribute to hepatocyte injury during MASH progression (Xiong et al., 2022; Yu and Song, 2024). Moreover, several studies have suggested that inhibition of ferroptosis induced by lipid metabolic disorders may represent a potential strategy for improving MASH (Cheng et al., 2023; Zhao et al., 2025). The xCT/GPX4 axis is a central antioxidant defense system against ferroptosis. Iron overload and lipotoxic stress can impair this axis, leading to reduced xCT-mediated cystine uptake, decreased GSH availability, and diminished GPX4-dependent lipid peroxide detoxification, thereby aggravating lipid peroxidation, oxidative stress, and ferroptotic cell death (Lv et al., 2024). In the present study, increased Fe^2+^, lipid peroxide, and MDA levels in the liver tissues of HFFD-induced MASH mice indicated the activation of ferroptosis-related injury. WOG administration markedly reduced Fe^2+^, lipid peroxide, and MDA accumulation and increased hepatic xCT and GPX4 protein expression. Similar anti-ferroptotic effects were also observed in FFA-induced HepG2 cells. To further confirm the inhibitory effect of WOG on ferroptosis, FFA-induced HepG2 cells were treated with erastin, a ferroptosis inducer, or Ferrostatin-1, a ferroptosis inhibitor. WOG inhibited erastin-induced increases in lipid peroxide and Fe^2+^ levels and reversed the erastin-mediated downregulation of xCT and GPX4 expression. Conversely, WOG exerted effects similar to those of Ferrostatin-1 by reducing cellular lipid peroxide and Fe^2+^ levels and upregulating xCT and GPX4 expression. These findings suggest that activation of the xCT/GPX4 axis is an important mechanism by which WOG inhibits ferroptosis and alleviates MASH-related hepatocellular injury. In addition, ferroptosis and inflammation may reinforce each other during MASH progression. Hepatic iron overload and ferroptotic injury can activate macrophage-mediated inflammatory responses, while inflammatory cytokines may further aggravate hepatocyte injury and lipid metabolic dysfunction (Van den Bossche et al., 2017; Wang B. et al., 2023). In this study, WOG reduced the expression of pro-inflammatory cytokine genes, including *TNF-α*, *IL-6*, and *IL-1β*, in macrophages, including RAW264.7 cells and Kupffer cells, suggesting that WOG may also attenuate macrophage-associated inflammatory amplification in MASH. These findings suggest that WOG suppresses ferroptosis, at least in part, by restoring xCT/GPX4-mediated antioxidant defense. Together with AMPK-mediated metabolic reprogramming, this anti-ferroptotic effect may help interrupt the pathological cycle of lipid accumulation, oxidative stress, inflammation, and hepatocyte injury in MASH.

Taken together, our findings demonstrate that WOG exerts protective effects against MASH through the coordinated regulation of lipid metabolic dysfunction, oxidative stress, inflammation, and ferroptosis. On the one hand, WOG activated the AMPK signaling pathway, which suppressed SREBP-1c-mediated lipogenesis and enhanced PGC-1α/PPARα/CPT-1-related fatty acid *β*-oxidation, thereby reducing hepatic lipid accumulation and the availability of peroxidation-prone lipid substrates. On the other hand, WOG restored xCT/GPX4-mediated antioxidant defense, leading to reduced Fe^2+^ accumulation, lipid peroxidation, oxidative injury, and ferroptosis-related hepatocellular damage. These effects may jointly interrupt the pathological cycle of lipid overload, mitochondrial dysfunction, ROS accumulation, ferroptosis, and inflammatory amplification during MASH progression.

Nevertheless, several limitations should be acknowledged. Although the HFFD-induced model recapitulates key metabolic and histological features of human MASH, including metabolic stress, hepatic steatosis, inflammation, oxidative injury, and liver damage, it may not fully reflect the complex etiology, inter-individual heterogeneity, or fibrosis progression observed in patients. In addition, the present *in vitro* experiments used FFA-induced HepG2 cells and LPS-stimulated RAW264.7 macrophages/Kupffer cells as separate monoculture models. Although these models are widely used to evaluate hepatocellular steatosis/ferroptosis and macrophage inflammatory activation, they cannot fully mimic the lipotoxicity-driven sterile inflammation and hepatocyte–macrophage/Kupffer cell crosstalk characteristic of MASH. Given that WOG suppressed inflammatory activation in macrophages/Kupffer cells and reduced hepatic TNF-α expression *in vivo*, its protective effects may also involve modulation of the local hepatic immune microenvironment. Future studies using steatotic hepatocyte–macrophage/Kupffer cell co-culture systems or conditioned media from FFA-treated hepatocytes will help determine whether WOG regulates intercellular communication and inflammatory amplification during MASH progression. Furthermore, although the WOG doses used in this study were effective in mice, their translational relevance requires further consideration. Based on body surface area normalization, the mouse doses of 2.5, 5, and 10 mg/kg correspond to approximate human equivalent doses of 0.20, 0.41, and 0.81 mg/kg, respectively, equivalent to approximately 12, 24.6, and 48.6 mg/day for a 60-kg adult. However, direct extrapolation from mice to humans should be interpreted cautiously because oral bioavailability, metabolism, systemic exposure, tissue distribution, and long-term safety may differ substantially between species. Further studies using pathway-specific inhibitors, gene knockdown models, additional preclinical MASH models, human-derived cellular systems, and clinical samples are warranted to clarify the causal relationship between AMPK activation and xCT/GPX4-mediated ferroptosis inhibition. Dedicated pharmacokinetic, toxicological, and clinical studies are also needed to determine whether therapeutically effective WOG exposure can be safely achieved in humans.

## 5 Conclusion

**FIGURE 8:**
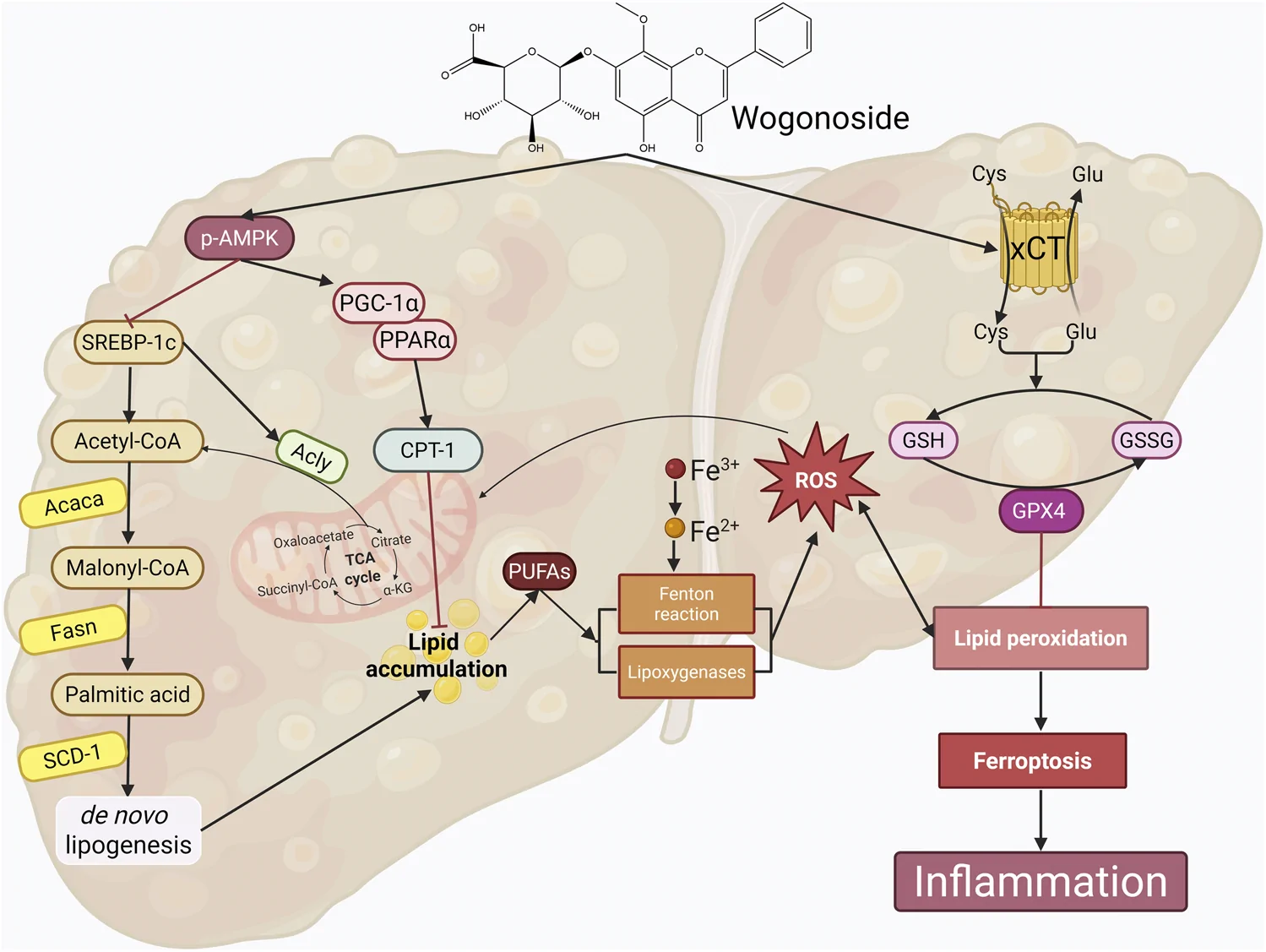
Schematic diagram showing the mechanism of the improved effect of WOG on MASH.

Overall, our study demonstrates that WOG effectively ameliorates MASH in HFFD-fed mice. The present findings suggest that WOG exerts anti-MASH effects through two closely interconnected mechanisms. First, WOG restores the xCT/GPX4 pathway to suppress ferroptosis, thereby reducing hepatic lipid peroxidation, oxidative stress, and inflammatory responses. Second, WOG activates AMPK signaling to inhibit lipid synthesis, promote fatty acid β-oxidation, and reduce hepatic lipid accumulation and the availability of polyunsaturated fatty acids, which serve as key substrates for ferroptosis (Figure 8). Collectively, these findings indicate that WOG may be a promising therapeutic candidate for MASH and provide new mechanistic insight into its potential clinical application.

## Data Availability

The data presented in the study are deposited in the NCBI BioProject repository, accession number PRJNA1504979.
