# Supplementary material for: Wogonoside alleviates metabolic dysfunction-associated steatohepatitis by modulating AMPK and xCT/GPX4 pathways to restrain lipid dysregulation and ferroptosis
Source: Front Pharmacol. 2026 Aug 7;17:1852232. doi: 10.3389/fphar.2026.1852232 (PMC13494713; doi:10.3389/fphar.2026.1852232)
Supplement: Supplementary file 1 [file Supplementaryfile1.docx]

**Supplementary material**

**Wogonoside Alleviates Metabolic Dysfunction-associated Steatohepatitis by Modulating AMPK and xCT/GPX4 Pathways to Restrain Lipid Dysregulation and Ferroptosis**

**1 Methods**

1.1 Cell cultures and cytotoxicity assay

HepG2 cells and Raw264.7 cells were cultured in DMEM medium containing 10% FBS, while Kupffer cells were cultured in RPMI 1640 medium containing 10% FBS. Frozen cells were thawed from a -80°C freezer and cultured in a 37°C incubator with 5% CO₂ until the cell confluence reached 80–90%, at which point they were passaged. HepG2, Raw264.7, and Kupffer cells in the logarithmic growth phase were seeded at a density of 1 × 10⁴ cells per well into a 96-well plate. After the cells adhered, drugs at final concentrations of 2.5 μM, 5 μM, and 10 μM were added to assess drug toxicity. After 48 hours of treatment with different concentrations of WOG, add 10 μL of MTT solution to each well and incubate for 4 hours. Remove the supernatant, add 160 μL of DMSO solution to each well, and shake at room temperature in the dark for 30 minutes. Measure the absorbance at 490 nm using a microplate reader and calculate the cell survival rate.

1.2 Detection of NO content in RAW264.7 and Kupffer cells

RAW264.7 or Kupffer cells were seeded in 24-well plates at 1 × 10⁵ cells/well during the logarithmic growth phase. After 24 h, cells were pretreated with WOG (2.5, 5, or 10 μM) or dexamethasone (DEX, 1 μg/mL) for 2 h, followed by co-stimulation with LPS (1 μg/mL) and the respective drugs for 24 h. The culture supernatant (50 μL) was mixed with 50 μL of Griess Reagent I and II sequentially. Absorbance was measured at 540 nm using a microplate reader.

Quantitative Real-Time PCR (qPCR) analysis of total RNA was extracted from treated RAW264.7 or Kupffer cells using TRIzol reagent. cDNA was synthesized using a PrimeScript RT Reagent Kit (Takara, Beijing, China). qPCR was performed using SYBR Green mix on a real-time PCR system. GAPDH was used as the internal control. Primer sequences are listed in Table S1.

Table S1. Primers and probes for real-time qRT-PCR

| Gene | Primer sequence (5′–3′) |
| --- | --- |
| TNF-α | Forward Primer： CCCTCACACTCAGATCATCTTCT  Reverse Primer： GCTACGACGTGGGCTACAG |
| IL-6 | Forward Primer： TAGTCCTTCCTACCCCAATTTCC  Reverse Primer： TTGGTCCTTAGCCACTCCTTC |
| IL-1β | Forward Primer： GAAATGCCACCTTTTGACAGTG  Reverse Primer： TGGATGCTCTCATCAGGACAG |
| GAPDH | Forward Primer： AGGTCGGTGTGAACGGATTTG  Reverse Primer： GGGGTCGTTGATGGCAACA |

1.3 Statistical analysis

All experimental data were statistically analyzed using SPSS 26.0 software (IBM SPSS, Chicago, IL, USA) and are presented as mean ± standard error. Data were analyzed using T-tests and one-way ANOVA, and *P* < 0.05 was considered statistically significant. The results were graphed using GraphPad Prism 8.0.2 software.

**2. Result**

2.1 Inhibitory effects of WOG on RAW264.7, Kupffer, and HepG2 cells

Preliminary in vivo experiments have confirmed that WOG has a positive effect on improving the progression of MASH. We will further analyze the therapeutic effects of WOG in vitro. We used the MTT assay to analyze the inhibitory effects of WOG on RAW264.7, Kupffer, and HepG2 cells. As shown in Fig. S1 A-C, WOG had no inhibitory effect on any of the three cell types. Therefore, we selected a concentration with no inhibitory effect for subsequent experiments.

**Fig. S1.** Effect of WOG on cell viability. (A-C) Effect of different concentrations of WOG on cell viability of Raw264.7, Kupffer, and HepG2 cells

2.2 Effect of WOG on the inflammation response of LPS-induced macrophages

To further investigate the effects of WOG on the inflammatory response, we used LPS to stimulate Raw264.7 and Kupffer cells to establish an inflammatory cell model. Under continuous LPS stimulation, cells induce the production of large amounts of NO, and the generation of NO promotes the release of inflammatory factors and the sustained occurrence of the inflammatory response. After treating the cells with different concentrations of WOG, we measured the NO content in the cell culture supernatants of Raw264.7 and Kupffer cells. As shown in Fig. S2 A and E, the NO content in the cell culture supernatants significantly increased under LPS stimulation, while WOG intervention resulted in a concentration-dependent reduction in NO content in the cell culture supernatants. Notably, WOG at all concentrations exhibited a greater inhibitory effect on NO production than the positive control Dexamethasone (DEX).

Next, we examine the effects of WOG on the mRNA levels of inflammatory factors (TNF-α, IL-6, and IL-1β) by using q-PCR. Under LPS stimulation, the mRNA levels of *TNF-α*, *IL-6*, and *IL-1β* in Raw264.7 cells were significantly up-regulated by 4.9-fold, 12-fold, and 6.4-fold, respectively, while those in Kupffer cells were significantly up-regulated by 2.1-fold, 14.8-fold, and 2.0-fold, respectively (Fig. S2 B-D, F-H). Following WOG treatment, the mRNA levels of inflammatory factors (TNF-α, IL-6, and IL-1β) in both Raw264.7 and Kupffer cells were significantly reduced, with the best inhibitory effect observed at a concentration of 10 μM. These results indicate that WOG can reduce inflammation in cells by inhibiting the transcription of inflammatory factors.

**Fig. S2.** Effects of Wogonoside on inflammation. (A) NO content in the inflammation model of Raw264.7 cells. (B-D) The mRNA levels of TNF-α, IL-6, and IL-1β in the Raw264.7 cell inflammation model. (E) NO content in the inflammation model of Kupffer cells. (F-H) The mRNA levels of TNF-α, IL-6, and IL-1β in the Kupffer cell inflammation model. Experimental data have been expressed as mean ± standard deviation (n=3); ^###^P < 0.001 compared with the CN group; *P < 0.05, **P < 0.01, and ***P < 0.001 compared with the LPS group.
